# Investigation of anticorrosive behaviour of novel *tert*-butyl 4-[(4-methyl phenyl) carbonyl] piperazine-1-carboxylate for carbon steel in 1M HCl

**DOI:** 10.1016/j.heliyon.2021.e06090

**Published:** 2021-02-09

**Authors:** B.M. Praveen, B.M. Prasanna, N.M. Mallikarjuna, M.R. Jagadeesh, Narayana Hebbar, D. Rashmi

**Affiliations:** a Department of Chemistry, School of Engineering and Management, Srinivas University, Mukka, Mangalore, Karnataka, India; b Department of Chemistry, Jain Institute of Technology, Davangere, Viswesaraya Technological University, 577 003, Belagavi, Karnataka, India; c Department of Chemistry, Kuvempu University, Shankaraghatta, Shimoga, 577451, India; d Department of Physics, Jain Institute of Technology, Davangere, Viswesaraya Technological University, 577 003, Belagavi, Karnataka, India; e Department of Chemistry, SDM College Ujire, Dakshina Kannada, 574240, Viswesaraya Technological University, Belagavi, Karnataka, India

**Keywords:** TBMPCPC, Corrosion inhibition, Electrochemical, SE

## Abstract

The main focus of current research is on the synthesis and anticorrosive activity of novel heterocyclic compounds tert-butyl 4-[(4-methyl phenyl) carbonyl] piperazine-1-carboxylate [TBMPCPC]. Electrochemical, quantum chemical, and surface characterization studies at elevated temperatures (303–333 K) for carbon steel in 1M HCl solution studied this molecule's corrosion inhibition property. It is observed from the results of electrochemical studies that the TBMPCPC may be able to effectively protect the steel plate surface with an inhibition efficiency of 91.5 % at 25 ppm in corrosive media. The corrosion inhibition depends on concentration, as concentration also increases inhibition efficiency due to the strong and spontaneous adsorption on the metal's surface. The Tafel polarization measurements concluded that the inhibitor works as a mixed form to protect the carbon steel in the bulk solution from corrosion. The adsorption of the TBMPCPC onto the metal surface was in reliable with the isothermal model of the Langmuir adsorption. The scanning electron microscopy clearly showed that the inhibitor was substantially deposited on the metal surface, indicating substantial inhibition. The surface morphology of carbon steel in the absence and existence of an inhibitor in 1 M HCl is also studied using the Atomic Force Microscopic method.

## 1 Introduction

Carbon steel is a crucial iron alloy and is primarily used in many fields of industry and construction. In general, carbon steel is used due to its high thermal stability and excellent mechanical properties in the petroleum industry, storage containers, and reaction vessels. In these areas, metal surfaces in pickling, decaling, acid washing, and other applications [1, 2] are exposed to aggressive media, causing corrosion, resulting in the loss of the metal content of the devices as described above. Metal loss can be avoided by using the most convenient and acceptable methods for corrosion control. Among these, the most effective and timely technique for studying the effect of corrosion inhibition on carbon steel in acidic media is to use corrosion inhibitors.

Corrosion inhibitors are derived predominantly from heterocyclic compounds containing atoms rich in electrons such as N, S, O, and π-electrons [3, 4]. Because of the formation of a protective coating on the metal's surface, the inhibitor molecules prevent metal corrosion. Palm oil [5], Luffa cylindrical leaf [6], Apricot juice [7], Aloe vera gel [8], drug intermediates viz., cefuroxime [9], Cefazolin [10], hydralazine hydrochloride [11], Praziquantel [12] Schiff bases, azo dyes such as Thiadiazole-Derived Bis-Schiff Bases [13], N-(Benzo[d]thiazole-2-yl)-1-phenylethan-1-imines [14], Mono Azo Dyes derived from 4,5,6,7-Tetrahydro 1,3 benzothiazole [15], Most of the inhibitors are commercially available. Their inhibition performance has been improved at room temperature. However, with increasing temperatures, it decreases and is often toxic because of certain poisonous chromium and cyanide groups. Therefore, due to the absence of a chromium or cyanide group in the molecule, our chosen new urea derivative is non-toxic. And it also serves as an effective corrosion inhibitor for carbon steel in 1M HCl. The corrosion parameters were discussed using electrochemical experiments and surface morphology; the corrosion parameters were examined.

## 2 Experimental

### 2.1 Material

For the corrosion inhibition calculation, the commercial carbon steel strip with dimensions of 5 × 1 × 0.5 cm3 was used and mechanically abraded with SIC emery paper with high-grade number 2000 until we find a smooth and mirror finish. The soft, finished carbon steel strip was treated with acetone, washed with triple distilled water, and processed for drying in the desiccator. AR grade HCl in de-ionized water was prepared from the corrosive media of 1M HCl.

### 2.2 Inhibitor

The novel tert-butyl 4-[(4-methyl phenyl) carbam oyl] piperazine-1-carboxylate (TBMPCPC) heterocyclic molecule derived from the 4-methyl phenyl isocyanate and N-Boc piperazine reaction was our primary target compound to be used in corrosion inhibition measurements, in which it serves as a corrosive media inhibitor. The TBMPCPC inhibitor was first dissolved in 1 cm^3^ DMF and then poured into 1M HCl corrosive media at elevated 5–25 ppm concentrations. All corrosion studies have used these prepared inhibited solutions with different concentrations. As per the procedure explained below, the molecule was designed.

#### 2.2.1 Synthesis of tert-butyl 4-[(4-methyl phenyl) carbam oyl] piperazine-1-carboxylate (TBMPCPC)

**Scheme 1:**
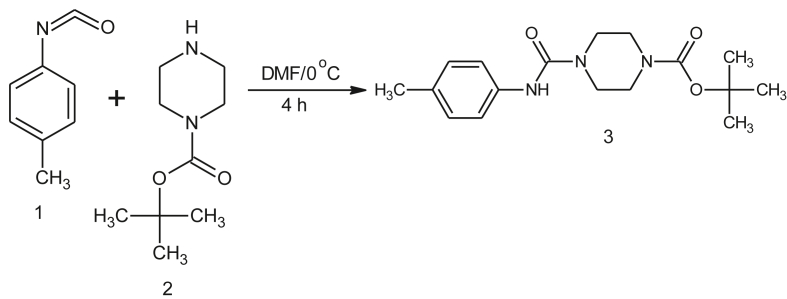
Synthesis of *tert*-butyl 4- [(4-methyl phenyl) carbanoyl] piperazine-1-carboxylate (TBMPCPC).

4-methyl phenyl isocyanate (1) was mixed at 0 °C with N-Boc piperazine cold solution (2) in DMF solvent and stirred for an hour. To supply the solid product, the above solution was treated with the ice-cold solution and filtered off, washed with triple distilled water, and dried to obtain the final product as piperazine-1-carboxylate tert-butyl 4-[(4-methyl phenyl) carbamoyl] (3). The schematic representation of the final compound synthesis (3) is provided in Scheme 1.

### 2.3 Structural characterization of the TBMPCPC

The target compound synthesised in the above scheme is white coloured amorphous solid and has the melting point in the region. It is completely soluble in typical organic solvents including DMSO, CdCl_3_ etc.

The compound 3 FT-IR spectrum showed a large peak in the region of 3200–3300 cm^−1^ due to the presence of the amide NH group. At 1767 and 1593 cm^−1^, the two carbonyl groups present in the compound exhibited a sharp peak. Because of the stretching vibrations of the C–C single and double bonds, a sharp peak appeared at 1123 cm^−1^. The compound's 1H NMR spectrum was reported as an internal standard reference in a DMSO-d6 solvent at room temperature with tetra methyl silane (TMS). The aromatic protons appeared in the region of 7.26–7.07 ppm as multiplets in the 1H NMR spectrum of the compound. The amide NH proton present in the molecule as singlet was reasoned at 4.05 ppm. The methyl groups present in the molecule were resonated at 1.45 ppm as singlets. Signals were seen in the range of 3.96–2.00 ppm for the remaining aliphatic protons. In addition, the compound's LCMS spectrum (3) showed a molecular ion peak at m/z 320, which is equal to its 319.39 molecular weight. It is therefore assumed from the above spectral data that all of these spectral data endorse the proposed molecular structure of the synthesised compound.

### 2.4 FTIR spectral characterisation

Using the Frontier PerkinElmer spectrometer, the FTIR spectrum for pure compound and the compound scarped from the carbon steel surface after corrosion were performed.

### 2.5 Electrochemical measurements

For all corrosion experiments, a traditional 3 electrode device was plugged into the CHI608D electrochemical workstation for the electrochemical experiments. The three electrode device consisted of a working electrode carbon steel strip, a counter electrode platinum electrode and a saturated calomel electrode as a reference electrode. For electrochemical measurements, a polished working electrode with an exposed area of 1 cm^2^ (the remainder of the portion was covered by epoxy resin) was used.

The carbon steel (working electrode) was dipped in the sample solution for about 30 min for the open circuit potential (OCP) to enter the steady state before performing the electrochemical experiment. At a scan rate of 0.01 mV/s, the potentiodynamic polarisation curves were obtained within the potential range of +0.2 to -0.2 for the OCP. Spectroscopic electrochemical impedance measurements were performed using AC signals with amplitude of 5 mV/s and a frequency range of 0.1–10 k Hz. By using specialised software Z-simp 3.21, all impedance information obtained from Nyquist's plots was fitted into an analogous circuit.

### 2.6 Thermodynamic parameters

The inhibitor's adsorption mechanism on the metal surface was studied using thermodynamic parameters that were evaluated from the precise isothermal adsorption model. Using EIS info, different adsorption isothermal models were designed to understand more about the TBMPCPC adsorption process on the steel surface in 1 M HCl solution at 303–333 K. For the evaluation of thermodynamic parameters which are associated with the inhibitory effect of the inhibitor, the most appropriate isotherm was chosen.

### 2.7 Atomic force spectroscopic (AFM) measurement

Atomic Force Microscopic (AFM) measurement was conducted for the surface investigation of carbon steel in the presence and absence of the 1M HCl inhibitor. In the absence and presence (25 ppm) of TBMPCPC for around 12 h, the carbon steels were immersed in 10 ml of HCl solution. The dipped steel strips were extracted from the water-washed solution after this time, dried and used for surface characterisation. In the images, the scanning area was 10 × 10 mm^2^.

### 2.8 Scanning electron microscopic (SEM) measurement

In the absence and presence of an optimised concentration of 25 ppm TBMPCPC in 1M HCl, the surface morphology of carbon steel strips was performed using SEM measurements with dimensions of 1 cm2. Using VEGA3 TESCAN SEM, SEM micrographs of steel strips immersed in 1 M HCl solution without and with TBMPCPC were reported at an accelerating beam of 25 kV.

## 3 Results and discussions

### 3.1 ^1^H NMR spectroscopy

**Figure 1:**
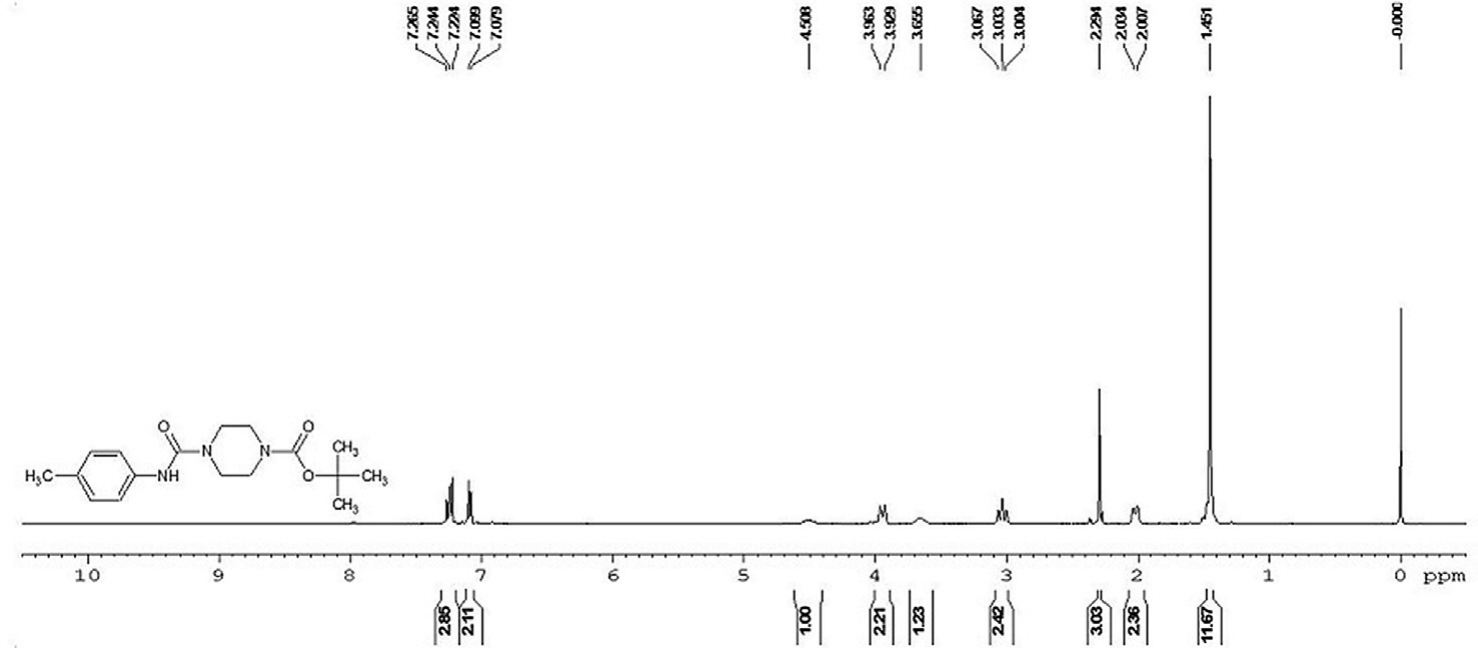
^1^H NMR spectra of tert-butyl 4- [(4-methyl phenyl) carbanoyl] piperazine-1-carboxylate.

Primarily the compound was confirmed by 1H NMR spectroscopy as shown in Figure 1. The compound having total 23 protons. In the range from 7.0 to 7.4 δ ppm, where aromatic appears in four protons. The proton NH oscillates and appears at 4.7 δ ppm. And eight cyclic methylene (-CH_2_) protons are appeared in the range 2–4 δ ppm a sharp and high intense peak at 1.5 δ ppm is assigned for 12 methyl (-CH_3_) protons. In Figure 1, the ^1^H NMR spectrum of the synthesized compound is shown.

### 3.2 FTIR studies

**Figure 2:**
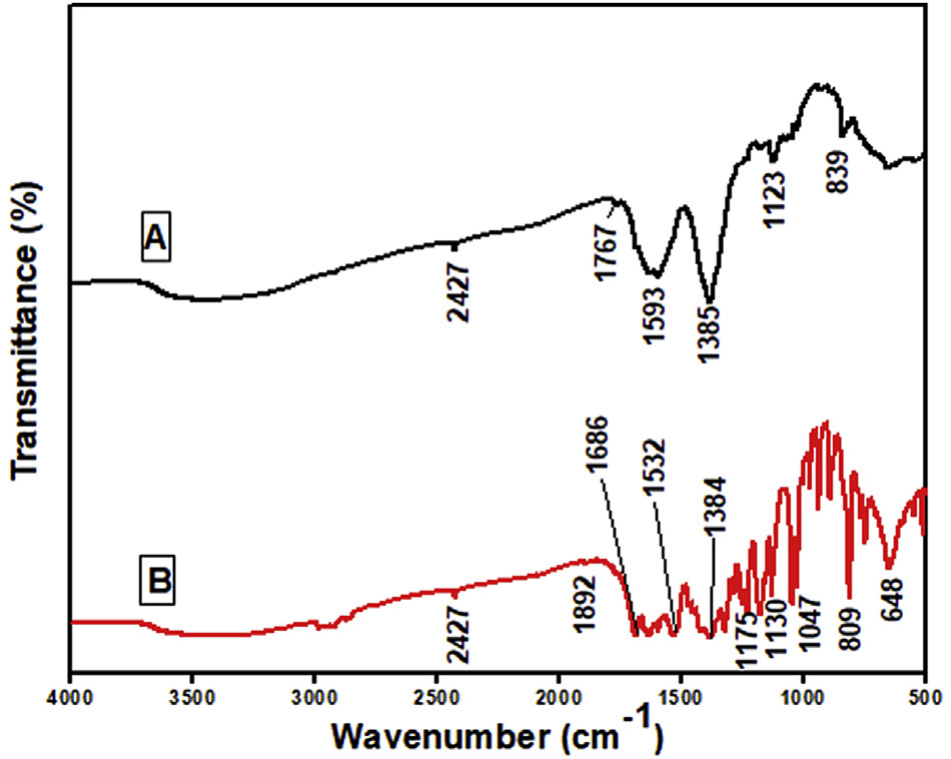
A) FTIR spectrum for pure Drug and B) Spectrum for scrapped TBMPCPC.

FTIR studies have analyzed the structural properties of the studied TBMPCPC and that of the commodity after scrapping from the carbon steel surface that was kept in contact with a 1M HCl solution containing a 25 ppm TBMPCPC inhibitor and shown in Figure 2 (A & B). The anticorrosive activity of the molecule on the surface of the carbon steel is determined and is due to the adsorption of the inhibitor to the surface of the steel by the interaction of the inhibitor's heteroatoms, which is confirmed by the peak shift in the pure drug and scrapped product IR spectra. At 1532 cm^−1^ of the scrapped compound, the absorption peak corresponds to the aromatic C=C bending vibration compared to 1593 cm^−1^ for the pure compound. The band for pure compound and scrapped compound at 1385 cm^−1^ and 1384 cm^−1^ corresponds to the absorption of C–H. The presence of a narrow peak for both the pure drug molecule and the scrapped compound at 1123 cm^−1^ and 1130 cm^−1^ confirms the absorption of aliphatic amines through C–N stretching.

### 3.3 Potentiodynamic tafel polarisation measurements

**Figure 3:**
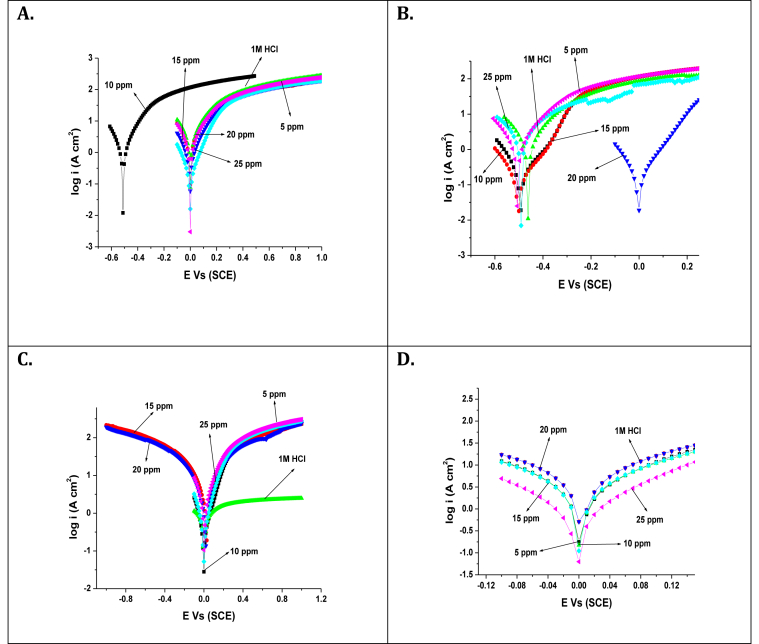
Tafel plots for carbon steel in the absence and presence of various concentrations TBMPCPC at (A) 303 K (B) 313 K (C) 323 K (D) 333 K temperatures.

The electrochemical Tafel polarisation curves for carbon steel without and with the addition of different concentrations of TBMPCPC in 1M HCl at 303–333 K were shown in Figure 3. The graph of current against potential was ploted at the given potential range at the scan rate of 0.01 mV/s. Table 1 consists of corrosion parameters viz., corrosion potential (E_corr_), corrosion current density (i_corr_), Corrosion rate (ν), Tafel cathodic slope (β_c_), Tafel anodic slope (β_a_) and inhibition efficiency (η_p_). The inhibition efficiency of TBMPCPC for carbon steel in 1M HCl is computed by the following expression [7],

(1) $$\eta_{p}=\frac{i^{0}-i}{i^{0}}\;\times 100$$

where, $i^{0}$ and irepresent the existing densities of corrosion without and with the inhibitor, respectively.

**Table 1:** Corrosion parameters found by Electrochemical Tafel polarization and Impedance spectroscopic measurements for carbon steel in the absence and presence of various concentrations TBMPCPC at (A) 303 K (B) 313 K (C) 323 K (D) 333 K.

Temperature (K)	Concentration of TBMPCPC (ppm)	Corrosion Potential E_corr_ (V)	Corrosion Current Density i_corr_ (A cm^−2^)	Corrosion Rate ν (mpy)	βc (mV/ decade)	βa (mV/ decade)	Inhibition Efficiency η_p_ (%)	Polarisation Resistance**Rp (*Ω cm^2^*)**	Cdl(μF)	Inhibition Efficiency (%) η_z_
303	1M HCl	-0.132	0.066	302.60	0.109	0.976	-	14.26	381	-
	5	-0.137	0.042	260.10	0.102	0.964	36.66	14.39	380	0.92
	10	-0.152	0.022	257.10	0.094	0.927	66.66	92.87	247	84.64
	15	-0.146	0.018	255.90	0.122	0.100	72.72	112.56	106	87.33
	20	-0.154	0.013	183.20	0.103	0.922	80.30	123.05	103	88.82
	25	-0.183	0.008	160.40	0.098	0.885	87.87	168.20	85	91.52
313	1M HCl	-0.621	0.029	327.4	0.116	1.361	-	17.46	274	-
	5	-0.646	0.023	276.00	0.096	0.939	20.68	27.72	247	37.01
	10	-0.681	0.017	270.50	0.096	0.855	41.37	64.32	191	72.85
	15	-0.711	0.015	265.20	0.099	0.839	48.27	92.87	121	81.19
	20	-0.193	0.014	202.20	0.097	0.824	51.72	125.55	84	86.47
	25	-0.568	0.006	174.20	0.107	0.621	79.31	159.77	83	89.07
323	1M HCl	-0.128	0.030	350.70	0.111	1.000	-	9.92	392	-
	5	-0.141	0.027	305.10	0.950	0.101	7.94	16.04	301	38.15
	10	-0.0485	0.025	292.10	0.884	0.985	16.88	17.52	290	43.37
	15	-0.050	0.023	276.20	0.957	1.052	22.84	42.71	149	76.77
	20	-0.174	0.019	223.80	0.101	0.881	36.09	46.26	133	78.55
	25	-0.174	0.016	192.50	0.099	0.881	46.68	70.52	115	85.93
333	1M HCl	-0.121	0.031	369.40	0.118	0.016	-	9.33	218	-
	5	-0.130	0.028	332.80	0.108	0.959	10.06	15.88	150	41.24
	10	-0.127	0.026	304.90	0.110	0.988	17.61	18.39	131	49.26
	15	-0.128	0.025	298.60	0.110	0.990	19.18	20.67	123	54.86
	20	-0.158	0.020	236.00	0.107	0.907	36.16	27.56	98	66.14
	25	-0.159	0.018	218.70	0.108	0.892	40.88	27.56	72	66.14

The results show that with increasing TBMPCPC concentrations, the corrosion rate is gradually decreasing. This is due to the accumulation of TBMPCPC by adsorption from the bulk solution onto the metal surface. The adsorbed molecules on metal surfaces block the corrosion sites, which reduces the rate of corrosion. It is also stated that the inhibited and uninhibited solution E_corr_ does not exceed ± 85 mV, indicating the action of the mixed form. This is the suppression of both anodic (i.e. metal dissolution) and cathodic (i.e. hydrogen release) reactions, resulting in a decrease in the rate of corrosion [16, 17]. The increasing inhibitory effect of the carbon steel inhibitor is also represented by the decrease in corrosion current density (i_corr_) and corrosion rate (v) with the increasing concentration of inhibitor in 1M HCl.

From the above data, it is concluded that the corrosion inhibition efficiency also increases for carbon steel in 1M HCl solution as the concentration of the inhibitor increases. The major change in the cathodic tafel slope (βc) and anodic tafel slope (βa) indicates that in our case, the selected inhibitor prevents corrosion by means of adsorption without altering the corrosion reaction mechanism.

Inhibitors typically display high efficiency of inhibition at room temperature, but at an elevated temperature, it decreases. Therefore, TBMPCPC exhibits high inhibition efficiency, but due to the destruction of adsorbed layers over the carbon steel surfaces, inhibition efficiency decreases with the increasing temperature.

### 3.4 Electrochemical impedance spectroscopic (EIS) measurements

**Figure 4:**
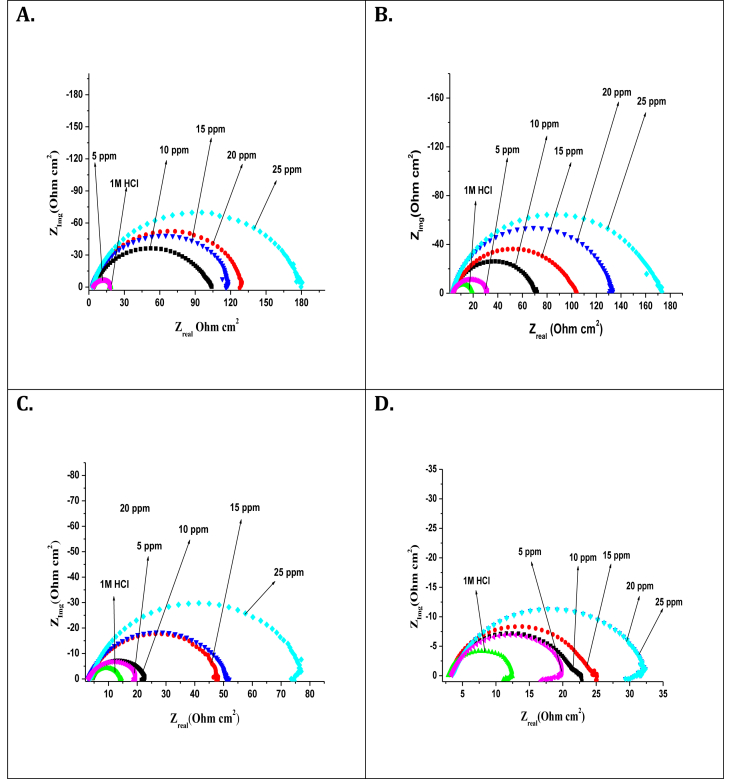
Nyquist's plot plots for carbon steel in the absence and presence of various concentrations of TBMPCPC at (A) 303 K (B) 313 K (C) 323 K (D) 333 K.

**Figure 5:**
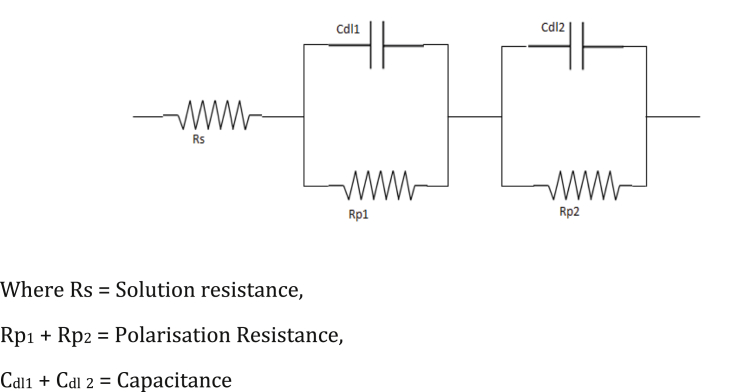
Equivalent circuit for fitting EIS data.

EIS measurements have investigated the anticorrosive potential of carbon steel in the absence and presence of TBMPCPC in 1 M HCl at 303–333 K. From this, Nyquist's plots (Figure 4) were obtained using 5 mV/s amplitude AC signals with a frequency range of 10 kHz. Z-simp 3.21 software fitted the electrochemical data obtained from the Nyquist's plots into an analogous circuit as shown in Figure 5.

**Figure 6:**
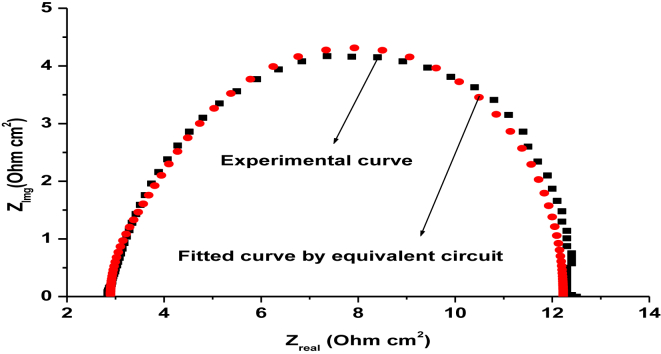
The fitted curve found by the electrical equivalent circuit for a sample Nyquist's plot.

The resulting corrosion parameters have been listed in Table 1, such as polarization resistance (Rp), capacitance (C_dl_). As shown in Figure 6, the sample experimental curve was precisely equipped with the curve defined by the electrical equivalent circuit.

The inhibition efficiency (*ƞ*_*z*_) of the carbon steel inhibitor was achieved by the following equation.

(2) $$\eta_{z}=\frac{R_{P}^{0}-R_{P}}{R_{P}}\times 100$$

where $R_{P}^{0}$ and $R_{p}$ are the polarisation resistances of uninhibited and inhibited solution respectively. And also by the Helmholtz equation, the values of C_dl_ is given by the following equation,

(3) $$C_{dl}=\frac{\varepsilon \varepsilon_{0}A}{\delta }$$

where ε is the dielectric constant, $\varepsilon_{0}$ is the vacuum permittivity, *A* is the effective area of the electrode and δ is the thickness of the protective layer.

The corrosion parameters measured from the EIS measurements indicate that the increased concentration of TBMPCPC in 1M HCl increases the diameter of the semicircles in the plot of Nyquist. This is due to the adsorption of the inhibitor to the surfaces of carbon steel from a bulk of 1M HCl solution. R_P_ values for inhibited solution with respect to uninhibited solution increase, resulting in an increase in inhibition performance of the carbon steel inhibitor. The inhibitor effectively decreases carbon steel corrosion by increasing the concentration of TBMPCPC in 1M HCl solution. Owing to the adsorption of the inhibitor on the surfaces of the carbon steel from the bulk of the solution, a maximum inhibition efficiency of around 91.50 % was observed for optimized inhibitor concentrations of 25 ppm.

However when at 303–333 K, the temperature of the corrosive media increases, the values of ƞ_z_ decreases due to the superiority of the mechanism of desorption over the inhibitor adsorption on the metal surfaces. The adsorbed layer that decreases the inhibition efficiency of TBMPCPC for carbon steel in 1 M HCl can be decreased by increasing temperature.

Another important corrosion parameter found by the EIS calculation is the C_dl_ value, which also defines the anticorrosive nature of the 1M HCl steel corrosion inhibitor. The decrease in the C_dl_ value with increasing TBMPCPC concentrations in 1M HCl indicates that the increase in the thickness of the double layer by adsorption of TBMPCPC in 1M HCl solution to the steel surface [18, 19]. Therefore it can be easily understood from this that our tested compound is capable of inhibiting the corrosion of the aggressive solution of carbon steel.

### 3.5 Thermodynamic consideration

**Figure 7:**
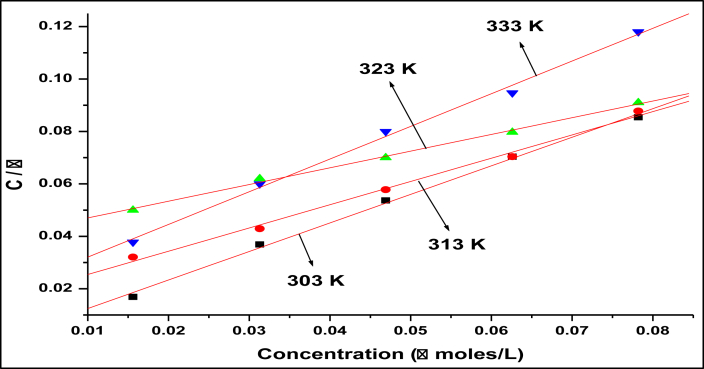
Langmuir's adsorption isotherm model.

In general, corrosion inhibitors have been identified in acid media to protect the corrosion of metals by adsorbing themselves onto the metal surface [20]. In order to know more details about the mode of adsorption attempts to fit data discovered from experiments into different models of adsorption isotherm viz., Langmuir, Freundlich, Temkin, Frumkin etc the best fit results observed near unity with regression coefficient (R^2^), the adsorption isotherm of Langmuir for this analysis, which is the strong agreement with the following expression.

(4) $$\frac{C}{\theta }=\frac{1}{K_{ads}}+C$$

where C is TBMPCPC concentration, the adsorption equilibrium constant is K_ads_. The Langmuir isothermal adsorption model is shown in Figure 7 for TBMPCPC at 303–333 K on the carbon steel surface. In Table 2, the corrosion parameters found in the adsorption studies were stated.

**Table 2:** Thermodynamic and adsorption parameters for carbon steel without and with the addition of TBMPCPC in 1M HCl at 303–333 K.

Temperature (K)	*K*_*ads*_ (kJ mol^−1^)	*ΔG*^*0*^_*ads*_ (kJ mol^−1^)	*ΔH*^*0*^_*ads*_ (kJ mol^−1^)	*ΔS*^*0*^_*ads*_ (kJ mol^−1^)
303	64935	-38.03	-103	-0.214
313	60606	-39.10		-0.204
323	51020	-41.13		-0.185
333	24,630	-37.93		-0.201

The set of straight lines was discovered with the regression coefficient almost reaching unity on Langmuir's adsorption isotherm map. The values of K_ads_ were calculated with the aid of slope values. The following expression measured the free energy shift of adsorption ΔG^0^_ads_ of the inhibitor on steel surfaces as,

(5) $$\Delta G_{ads}^{0}=-RT\;ln\;\left(K_{ads}\times 55.5\right)$$

Where *ΔG*^*0*^_*ads*_ is the normal free adsorption energy shift, K_ads_ is the equilibrium constant for the inhibitor adsorption to the steel surface and 55.5 is the solution concentration of water expressed in mol/L terms. The Kads and *ΔG*^*0*^_*ads*_ computed values are listed in Table 2. With the rise in temperature, the values of K_ads_ discovered from adsorption plots decrease. The adsorption of the inhibitor on the metal surfaces would be greater than the K_ads_ values.

The values of *ΔG*^*0*^_*ads*_ suggest that the adsorption of the inhibitor to the metal surface, which is approximately -20 kJ/mol, is presumed for physisorption and the chemisorption indication is approximately -40 kJ/mol. The negative indication of the importance of *ΔG*^*0*^_*ads*_ is an indication of the inhibitor's spontaneous adsorption on the carbon steel surfaces. The values of *ΔG*^*0*^_*ads*_ are around -40 kJ/mol, defining the chemisorption mechanism of inhibitor adsorption [21]. The findings therefore indicated that the inhibitor molecule is spontaneously adsorbed from 1M HCl solution to the surfaces of carbon steel.

The Gibbs-Helmholtz equation measures the enthalpy and entropy of adsorption ($\Delta H_{ads}^{0}$ and $\;\Delta S_{ads}^{0}$) as follows [22],

(6) $$\left(\frac{\Delta G/T}{\Delta T}\right)p=\frac{H}{T^{2}}$$

The rearranged form of the above equation is as follows,

(7) $$\Delta S_{ads}^{0}=\left(\Delta H_{ads}^{0}-\Delta G_{ads}^{0}\right)/\;T$$

The reordered form of the above equation is as follows.

**Figure 8:**
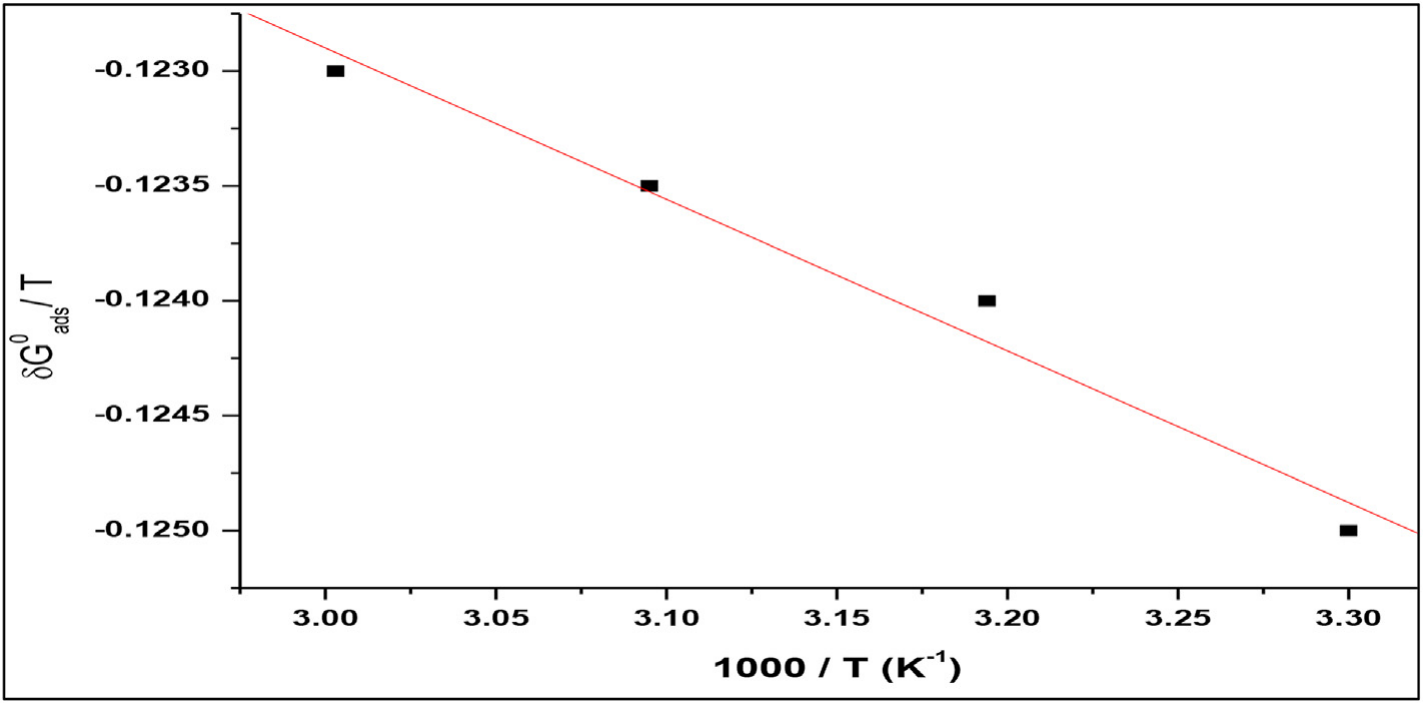
Plot of 1000/T versus $\Delta G_{ads}^{0}$/T.

Figure 8 describes a plot of $\Delta G_{ads}^{0}$/T against 1000/T with a slope value equal to $\Delta H_{ads}^{0}$. Generally, the values of $\Delta H_{ads}^{0}$ reach -100 kJ/mol include the process of chemisorption [23, 24]. In this work, we find that the magnitude of adsorption enthalpy is -103 kJ/mol indicates that the inhibitor has been chemically adsorbed on carbon steel surfaces. The adsorption entropy ($\Delta S_{ads}^{0}$) is also observed to decrease with rising temperature, and it is proposed that the inhibitor molecules are properly adsorbed to the carbon steel surfaces in 1M HCl solution [25].

### 3.6 Scanning electron microscopic (SEM) measurement

**Figure 9:**
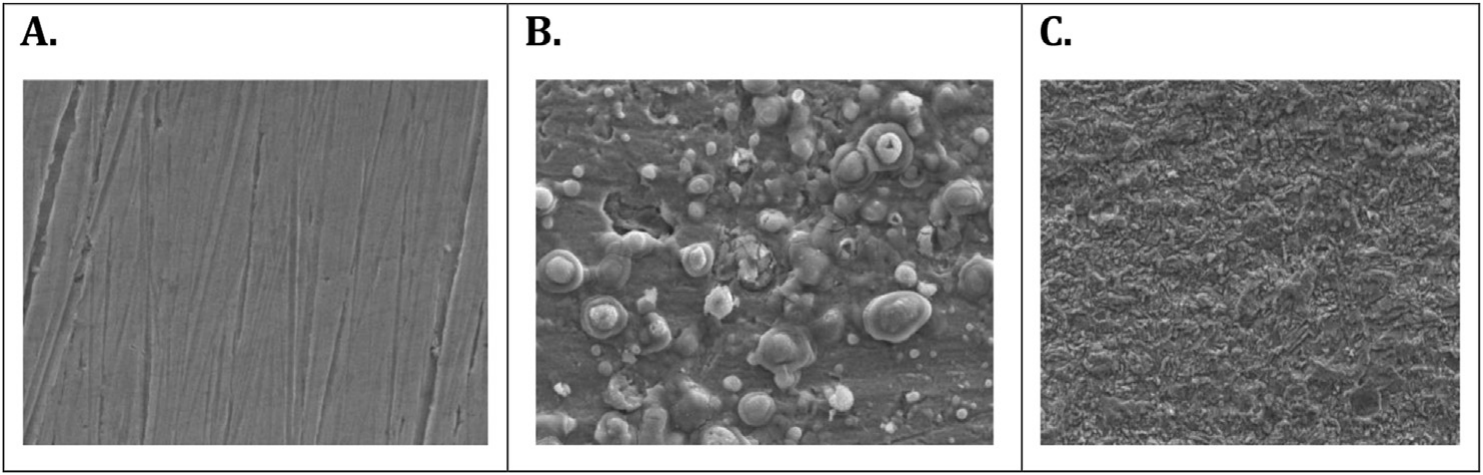
The SEM micrograph of (A) Polished carbon steel surface (B) Carbon steel surface in 1M HCl (C) Carbon steel surface dipped in 1M HCl with 50 ppm of TBMPCPC.

SEM has been studying the surface analysis of carbon steel. The SEM micrographs of the surfaces of carbon steel are shown in Figure 9. The polished carbon steel (Figure 9A) has smooth surfaces but has plenty of pits and corrosive materials on the same surfaces that are immersed in 1M HCl (Figure 9B). The carbon steel surfaces that were immersed with the addition of the inhibitor in 1M HCl (Figure 9C) showed that the TBMPCPC adsorbed layer on the surfaces of carbon steel. The deposited layer acts as a protective barrier for protecting carbon steel from corrosion by means of an inhibitor which delays corrosion [26].

### 3.7 Atomic Force Microscopic (AFM) measurement

**Figure 10:**
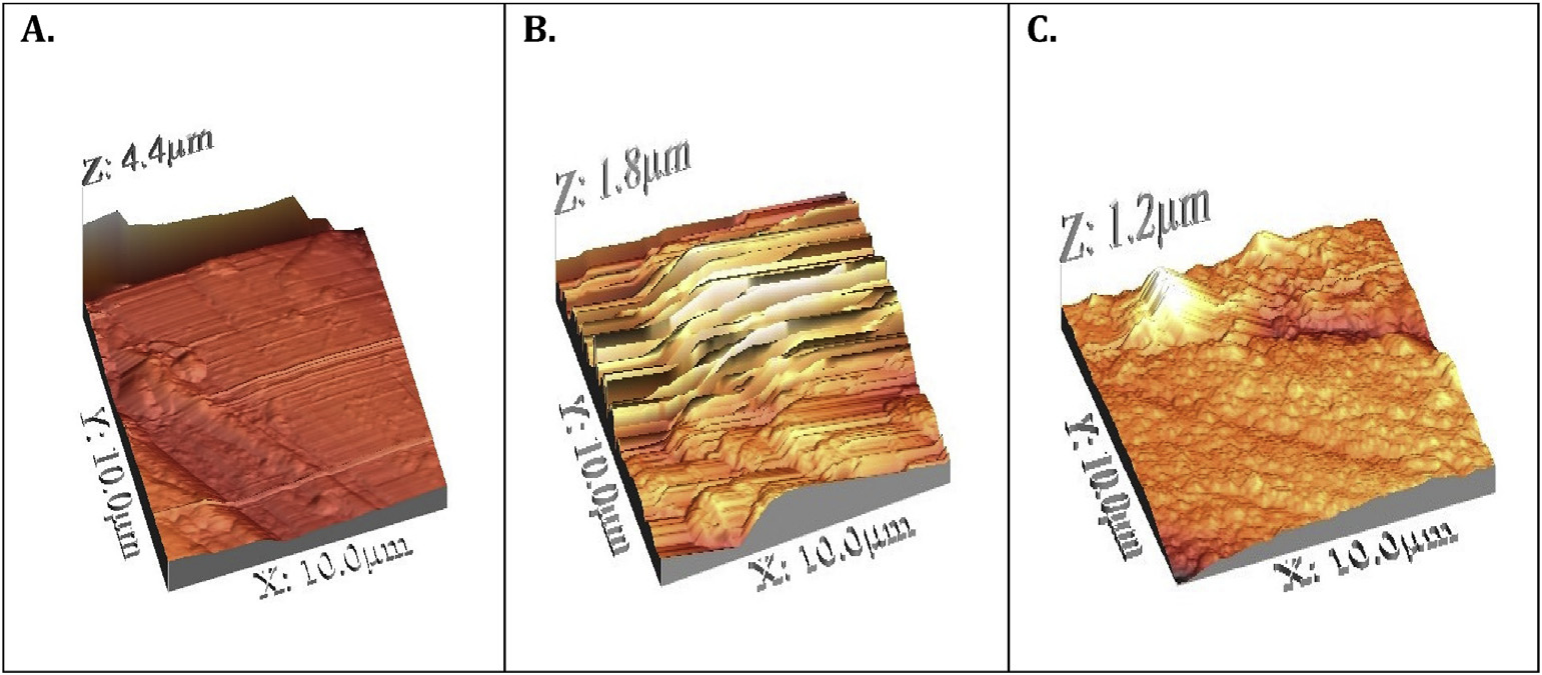
The AFM micrograph of (A) Polished carbon steel surface (B) Carbon steel surface in 1M HCl (C) Carbon steel surface dipped in 1M HCl with 50 ppm of TBMPCPC.

AFM is used to analyze nano-to-micro-scale surface morphology. This technique is useful in the study of the inhibitor's effect on metal surfaces by forming a protective barrier that prevents corrosion [27]. The AFM analysis provides information about the average roughness (R_a_) on the carbon steel surface that helps to assess the inhibitor's performance. The existence of the inhibitor adsorption on the surface of carbon steel can be explained on the basis of the R_a_ values (Figure 10).

In the absence and presence of an inhibitor, the 3D images and elevation profiles of polished carbon steel are shown in Figure 10 (A), the surface of polished carbon steel is clear of crack but exposed to 1 M HCl solution is affected by wide and deep crack as shown in Figure 10 (B). However in the case of carbon steel in contact with an inhibitor of 25 ppm, the carbon steel surface is deposited with a thin protective film, as shown in Figure 10(C), suggesting the adsorption of the corrosion-protecting inhibitor. The roughness parameter values also represent the process of inhibitor adsorption. The mean roughness (Ra) was found to be 4.4 μm for the polished carbon steel, while it was 1.8 μm for the polished carbon steel submerged in 1 M HCl for a duration of 12 h. From measurements on the surface of carbon steel submerged in 1 M HCl with an inhibitor of 25 ppm, the Ra value was found to be 1.2 μm, which is much lower than the blank value. In the presence of an inhibitor, the decrease in the Ra value shows the adsorption of inhibitor molecules to the surface of carbon steel [28].

## 4 Conclusions

• A good corrosion inhibitor for carbon steel in 1M HCl is found to be tert-butyl 4-[(4-methylphenyl) carbonyl] piperazine-1-carboxylate (TBMPCPC).
• The effectiveness of TBMPCPC inhibition increases with increasing concentration, but decreases with increasing inhibitor concentration.
• Inhibitory activity of the inhibitor due to the adsorption of inhibitor molecules from 1M HCl on the surfaces of carbon steel. The adsorption approach follows the adsorption isotherm model of Langmuir
• Adsorption parameters indicated that the inhibitor spontaneously adsorbed into carbon steel in 1M HCl solution via the primarily chemisorption process.
• SEM and AFM studies provide a visual concept for the creation of protective film by inhibitor adsorption, which reduces corrosion.

## Declarations

### Author contribution statement

Praveen B M: Conceived and designed the experiments.

Prasanna B M: Performed the experiments; Wrote the paper.

Mallikarjuna N M: Analyzed and interpreted the data.

Jagadeesh M R, Narayana Hebbar, Rashmi D: Contributed reagents, materials, analysis tools or data.

### Funding statement

This research did not receive any specific grant from funding agencies in the public, commercial, or not-for-profit sectors.

### Data availability statement

Data included in article/supplementary material/referenced in article.

### Declaration of interests statement

The authors declare no conflict of interest.

### Additional information

No additional information is available for this paper.

## References

[1] Fan H.L., Zheng H.B. (2002). Inhibition of mild steel in hydrochloric acid solution by a mercapto-triazole compound. J. S. Mater. Chem. Phys..

[2] Yadav M., Behera D., Sharma U. (2012). Nontoxic corrosion inhibitors for N80 steel in hydrochloric acid. Chem. Sin..

[3] Ahamad I., Quraishi M.A. (2009). Bis (benzimidazol-2-yl) disulphide: an efficient water soluble inhibitor for corrosion of mild steel in acid media. Corrosion Sci..

[4] Zhang Q.B., Hua Y.X. (2009). Carbon steel corrosion behavior in aqueous carbonated solution of MEA/[bmim] [DCA]. Electrochim. Acta.

[5] Carmona-Hernandez A., Vazquez-Velez E., Uruchurtu-Chavarin J., Gonzalez-Rodriguez J.G., Martinez-Gomez L. (2019). Use of an imidazole synthetized from palm oil as a corrosion inhibitor for a supermartensitic stainless steel in H_2_S. Green Chem. Lett. Rev..

[6] Ogunleye O.O., Arinkoola A.O., Eletta O.A., Agbede O.O., Osho Y.A., Morakinyo A.F., Hamed J.O. (2020). Green corrosion inhibition and adsorption characteristics of Luffa cylindrica leaf extract on mild steel in hydrochloric acid environment. Heliyon.

[7] Aprael S., Yaro Anees A., Khadom Rafal K., Wael (2019). Apricot juice as green corrosion inhibitor of mild steel in phosphoric acid. Alexandria Eng. J..

[8] Singh A.K., Mohapatra S., Pani B. (2016). Corrosion inhibition effect of Aloe Vera gel: gravimetric and 3 electrochemical study. J. Ind. Eng. Chem..

[9] Singh A.K., Quraishi M.A., Ebenso E.E. (2011). Inhibitive effect of Cefuroxime on the corrosion of mild steel in hydrochloric acid solution. Int. J. Electrochem. Sci..

[10] Singh A.K., Quraishi M.A. (2010). Effect of Cefazolin on the corrosion of mild steel in HCl solution. Corrosion Sci..

[11] Prasanna B.M., Praveen B.M., Narayana Hebbar, Venkatesha T.V. (2016). Experimental and theoretical studies of hydralazine hydrochloride as corrosion inhibitor for mild steel in HCl acid medium. Anti-corrosion Methods & Mater..

[12] Praveen B.M., Prasanna B.M., Narayana Hebbar, Shivakeshava Kumar P., Jagadeesh M.R. (2018). Experimental and theoretical studies on inhibition effect of the Praziquantel on mild steel corrosion in 1 M HCl. J. Bio. Tribo-Corrosion.

[13] Chugh Bhawna, Singh Ashish K., Thakur Sanjeeve, Pani Balaram, Lgaz Hassane, Chung Ill-Min, Jha Ranjana, Ebenso Eno E. (2020). Comparative investigation of corrosion-Mitigating behavior of thiadiazole-derived bis-Schiff bases for mild steel in acid medium. Experimental, theoretical, and surface study. ACS Omega..

[14] Chugh B., Singh A.K., Thakur S., Pani B., Pandey A.K., Lgaz H. (2019). An Exploration about the interaction of mild steel with hydrochloric acid in the presence of N-(Benzo[d]thiazole-2-yl)-1-phenylethan-1-imines. J. Phys. Chem. C.

[15] Mallikarjuna N.M., Keshavayya J., Prasanna B.M., Praveen B.M., Tandon H.C. (2019). Synthesis, characterization, and Anti-corrosion behavior of novel Mono azo dyes derived from 4,5,6,7-Tetrahydro-1,3-benzothiazole for mild steel in acid solution. J. Bio. Tribo-Corrosion.

[16] Ali S.A., El-Shareef A.M., Al-Ghandi R.F., Saeed M.T. (2005). The isoxazolidines: the effects of steric factor and hydrophobic chain length on the corrosion inhibition of mild steel in acidic medium. Corrosion Sci..

[17] Jayaperumal D. (2010). Effects of alcohol-based inhibitors of corrosion of mild steel in hydrochloric acid. Mater. Chem. Phys..

[18] Ferreira E.S., Giancomlli C., Giacomlli F.C., Spinelli A. (2004). Evaluation of the inhibitor effect of L-ascorbic acid on the corrosion of mild steel. Mater. Chem. Phys..

[19] Bentiss F., Lebrini M., Vezin H., Chai F., Traisnel M., Lagrenee M. (2009). Enhanced corrosion resistance of carbon steel in normal sulfuric acid medium by some macrocyclic polyether compounds containing a 1,3,4-thiadiazole moiety: AC impedance and computational studies. Corrosion Sci..

[20] Qu q., Jiang S.A., Bai W., Li L. (2007). Effect of ethylenediamine tetraacetic acid disodium on the corrosion of cold rolled steel in the presence of benzotriazole in hydrochloric acid. Electrochim. Acta.

[21] Badiea A.M., Mohana K.N. (2009). Effect of temperature and fluid velocity on corrosion mechanism of low carbon steel in presence of 2-hydrazino-4,7-dimethylbenzothiazole in industrial water medium. Corrosion Sci..

[22] Badr G.E. (2009). The role of some thiosemicarbazide derivatives as corrosion inhibitors for C-steel in acidic media. Corrosion Sci..

[23] Popova A., Sokolova E., Raicheva S., Christov M. (2003). AC and DC study of the temperature effect on mild steel corrosion in acid media in the presence of benzimidazole derivatives. Corrosion Sci..

[24] Obot I.B., Obi-Egbedi N.O., Eseola A.O. (2011). Anticorrosion potential of 2-Mesityl-1H-imidazo [4,5-f][1,10]-phenanthroline on mild steel in sulfuric acid solution: experimental and theoretical study. Ind. Eng. Chem. Res..

[25] Xianghong Li., Shuduan Deng., Hui Fu. (2011). Benzyltrimethylammonium iodide as a corrosion inhibitor for steel in phosphoric acid produced by dihydrate wet method process. Corrosion Sci..

[26] Matad Prasanna B., Mokshanatha Praveen B., Narayana Hebbar, Venkatesha Venkatarangaiah T., Harmesh Chander Tandon (2014). Ketosulfone drug as a green corrosion inhibitor for mild steel in acidic medium. Ind. Eng. Chem. Res..

[27] Abd E.A.F.S., Ali A.H. (2018). Egy-dronate drug as promising corrosion inhibitor of C-steel in aqueous medium. Zast. Mater..

[28] Horcas I., Fernandez R., Gomez-Rodriguez J.M., Colchero J., Gomez-Herrero, Baro A.M. (2007). WSXM., A software for scanning probe microscopy and a tool for nanotechnology. Rev. Sci. Instrum..

